# Grouped circular data in biology: advice for effectively implementing statistical procedures

**DOI:** 10.1007/s00265-020-02881-6

**Published:** 2020-07-20

**Authors:** Lukas Landler, Graeme D. Ruxton, E. Pascal Malkemper

**Affiliations:** 1 Institute of Zoology, University of Natural Resources and Life Sciences, Gregor-Mendel-Straße 33/I, 1180 Vienna, Austria; 2 School of Biology, University of St Andrews, St Andrews, KY16 9TH UK; 3 Max Planck Research Group Neurobiology of Magnetoreception, Center of Advanced European Studies and Research (CAESAR), Ludwig-Erhard-Allee 2, 53175 Bonn, Germany

**Keywords:** Rayleigh test, Rao’s spacing test, Hermans-Rasson test, Gini test, Type I error, Rounding error

## Abstract

**Abstract:**

The most common statistical procedure with a sample of circular data is to test the null hypothesis that points are spread uniformly around the circle without a preferred direction. An array of tests for this has been developed. However, these tests were designed for continuously distributed data, whereas often (e.g. due to limited precision of measurement techniques) collected data is aggregated into a set of discrete values (e.g. rounded to the nearest degree). This disparity can cause an uncontrolled increase in type I error rate, an effect that is particularly problematic for tests that are based on the distribution of arc lengths between adjacent points (such as the Rao spacing test). Here, we demonstrate that an easy-to-apply modification can correct this problem, and we recommend this modification when using any test, other than the Rayleigh test, of circular uniformity on aggregated data. We provide *R* functions for this modification for several commonly used tests. In addition, we tested the power of a recently proposed test, the Gini test. However, we concluded that it lacks sufficient increase in power to replace any of the tests already in common use. In conclusion, using any of the standard circular tests (except the Rayleigh test) without modifications on rounded/aggregated data, especially with larger sample sizes, will increase the proportion of false-positive results—but we demonstrate that a simple and general modification avoids this problem.

**Significance statement:**

Circular data are widespread across biological disciplines, e.g. in orientation studies or circadian rhythms. Often these data are rounded to the nearest 1–10 degrees. We have shown previously that this leads to an inflation of false-positive results when testing whether the data is significantly different from a random distribution using the Rao test. Here we present a modification that avoids this increase in false-positives for rounded data while retaining statistical power for a variety of tests. In sum, we provide comprehensive guidance on how best to test for departure from uniformity in non-continuous data.

**Electronic supplementary material:**

The online version of this article (10.1007/s00265-020-02881-6) contains supplementary material, which is available to authorized users.

## Introduction

In biology, many variables are recorded on scales that are cyclical rather than linear—the common examples of such are compass directions, angles, times of year and times of day. What these cyclical scales have in common is that the measurement scale has a natural repeating period to it, and data can often be effectively presented on the circumference of a circle. For example, certain neurons that respond to animal heading (head direction cells) in freely moving rats are an integral part of the animal’s neuronal compass in the brain (Taube 2007). In order to determine if such a cell indeed responds to direction, one needs to investigate data on a circular scale. In linear statistics, a cell firing with a peak around 0° and decreasing firing equally towards both sides (± 10°) might be divided into two skewed modes, each on the extremes of this linearized scale (e.g. from 350° to 359° and 0° to 10°). However, this would be a dangerous misinterpretation of the actual behaviour of the cell, which, in this example, fires in directions in only a single grouping around 0° following a von Mises distribution (the circular analogue of a normal distribution).

Therefore, such circular data needs different statistical treatment from linear measures (like height and weight), and a number of monographs are available on the statistical treatment of such circular data (e.g. Batschelet 1981; Fisher 1995; Mardia and Jupp 2000; Jammalamadaka and SenGupta 2001; Pewsey et al. 2013; Ley and Verdebout 2017). The most common procedure in circular statistics is testing to see if a sample of values suggests a deviation from being uniformly distributed around the circle. One issue here for the researcher seeking to test such a null hypothesis is that the commonly used tests were developed for data which are continuously distributed around the circle, whereas (e.g. because of limits to the precision of estimation techniques) often values are recorded in an aggregated form (i.e. rounded or grouped in bins). This means that, instead of being continuously distributed, the possible recorded values are restricted to a finite number *m* of values equally spaced around the circle. A recent survey by Humphreys and Ruxton (2017) found that commonly experienced values of *m* are 4, 8, 12 and 36; with 12 and 36 being particularly common (Freedman 1979). These values suggest that the assumption of a continuous distribution is often strongly violated.

We have recently offered some guidance on how to overcome this mismatch between the assumptions of the statistical tests and commonly collected real-life data. The most commonly used circular test is the Rayleigh test, and Humphreys and Ruxton (2017) argued that this test could be used on samples of grouped data. However, that study only investigated sample sizes up to 50; in the current analysis, we extend that to larger sample sizes. Another commonly used test is the Rao spacing test, and we recently demonstrated that in its standard form, this test failed to control type I error rate well when data were grouped (Landler et al. 2019). We offered a variant form of the test where small perturbations were added to recorded values in order to break any ties between data values and demonstrated that this modification led to considerably improved performance on grouped data (Landler et al. 2019). That study, however, only considered unimodal departures from uniformity. This limitation is important because the Rao spacing test is considered particularly attractive if multimodal departures are expected (e.g. Landler et al. 2018). Hence, here we explored the performance of both versions of this test in detecting multimodal departures from uniformity.

We also expand on the range of tests considered. Landler et al. (2018) compared the performance of a battery of alternative tests for detecting departures from circular uniformity with continuous data. There, we argued for increased consideration of a hitherto rarely used test due to Hermans and Rasson (1985; based on our publication this test has been added to the *R* package *CircMLE* (Fitak and Johnsen 2017)). Here we consider how that test functions when applied to grouped data. Finally, Tung and Jammalamadaka (2013) introduced a test based on the Gini mean difference in arc lengths between adjacent points in a sample that they argue is asymptotically more powerful than the Rao spacing test. Here we explore whether this test also suffers from the same lack of control of type I error that we observed in the Rao test and whether this control can be regained by the same modification. We further test the relative powers of the Rao spacing test and this Gini mean test. In addition, we test the performance of two other omnibus tests, the Watson and Kuiper test (see Batschelet 1981), and explore their behaviour on grouped data.

It could be argued that the question of uniformity of binned data around the circle does not need to be treated as a purely circular statistics problem. For example, one could employ a chi-squared test of the null hypothesis of uniformity across the bins (see, e.g. Batschelet 1981). We, therefore, compare the power of the chi-squared test with the circular statistic tests on binned data.

In summary, researchers exploring circular data routinely test the null hypothesis of uniformity. They also commonly have rounded and grouped (rather than continuously distributed) data. We show how common practice easily leads to high risk of false-positive results. Here we provide a more comprehensive and definitive guidance than any currently available on how best to test for departure from uniformity in non-continuous data.

## Methods

We consider six “standard” tests of the null hypothesis of circular uniformity, which we call the Rayleigh, Watson, Kuiper, Rao spacing, Gini and HR tests. In addition, we employ the chi-squared test (using the *R* function *chisq.test*) for all grouped data with a sample size larger than five, to test the null hypothesis of random distribution between groups (see Ruxton and Neuhäuser (2010) for an introduction to the literature on the potential unsuitability of this test with low sample sizes). The Rayleigh test is the most commonly applied test of this null hypothesis; see for example Batschelet (1981) for a full description. We use this implementation of the test provided by the function *rayleigh.test* within the *R* package *circular* (Agostinelli and Lund 2017). For the Watson and Kuiper test, we use the function *watson.test* and *kuiper.test*, respectively, both provided in *circular*. For the Rao spacing test, we use the function *rao.spacing.test* (package *circular*) for continuous data as well as the general simulation method described in Landler et al. (2019) and summarized as follows:

We define the test for *n* observations *ϕ*_*1*_,…, *ϕ*_*n*_ taken in radians such that the values lie within [0,2*π*). We further assume that the observations are ordered from smallest to largest: *ϕ*_*1*_ < *ϕ*_*2*_ < …. < *ϕ*_*n-1*_ < *ϕ*_*n*_. We can define the set of *n* arc lengths between neighbouring points as

$$ {T}_i=\left\{\begin{array}{c}{\phi}_{i+1}-{\phi}_i,\kern1em if\ i<n\\ {}2\pi -{\phi}_n+{\phi}_1\kern1.25em if\ i=n\end{array}\right.. $$

The test statistic *U* is the sum of the differences between the actual arc lengths and their expected values under uniformity (2*π*/*n*):

$$ U=0.5\sum \limits_{i=1}^n\left|{T}_i-\frac{2\pi }{n}\right|. $$

We generate *N*_*R*_ samples from a continuous uniform distribution, each with the same sample size as the original sample. For each of these simulated samples, we calculate the value of the test statistic and calculate the number *N*_*e*_ of these simulated samples that produce a test statistic value equal to or greater than the test statistic value for the original data. The estimated *p* value is then (*N*_*e*_ + 1)/(*N*_*R*_ + 1). In our simulations, we used *N*_*R*_ = 10,000. *P* value calculation by random permutation is a standard procedure in statistics and arguably goes back to Dwass (1957); this procedure is used throughout the paper for “tie-breaking” versions of tests (see below), as well as for all versions of the HR and Gini tests.

The Gini and HR tests are carried out in the same fashion, only the test statistics differ. For the Gini test, the test statistic is given in Tung and Jammalamadaka (2013) as

$$ {G}_n=\left(\frac{2}{n\left(n-1\right)}\right)\sum \limits_{i=1}^{n-1}\sum \limits_{j=i+1}^n\frac{1}{2}\left|n{T}_i-n{T}_j\right|. $$

For the HR test, a full description is given in Hermans and Rasson (1985) and Pycke (2010), but the test statistic is

$$ V=\left(\frac{1}{n}\right)\sum \limits_{i=1}^n\sum \limits_{j=1}^n\left(\left|\left|{\phi}_i-{\phi}_j\right|-\pi \right|-\frac{\pi }{2}-2.895\left(\left|\mathit{\sin}\left({\phi}_i-{\phi}_j\right)\right|-\frac{2}{\pi}\right)\right). $$

As well as the standard tests, we also consider a version of each of the tests (called the tie-breaking “TB” version), where small perturbations are added to all data points prior to the implementation of the test in order to induce tie-breaking in situations where grouping can produce identical values. Specifically, we added very small random perturbations selected independently from a von Mises distribution with mean zero to each data point in both our original sample and in the simulated samples for the tests that we evaluate by simulation. That is for data point *ϕ*_*i*_ we obtain a perturbation *ɛ*_*i*_ drawn from a von Mises distribution with mean zero and with concentration parameter *κ*. In our simulations, we used *κ* = 1000. The higher the value of *κ*, the more concentrated the distribution. A value should be chosen that is high enough that the perturbations are much smaller than the granularity of the imprecision. That is, if (e.g.) original values were obtained to the nearest 10 degrees, then a value of κ should be selected to ensure that almost all perturbations are less than 1 degree. We then calculate the value of the test statistics for the observed sample with added perturbations. Notice that if the sum of the original value and perturbation is outside of (0,2π), we add or subtract 2π (modulo 360) as required to correct this prior to implementing the test. All functions written to perform these TB versions of all tests, as well as the original version of the Gini test, are available in the supplementary material (Online Resource 2).

We consider two types of data in our simulation study, continuously distributed data and data restricted to 36 values spaced evenly around the circle (as would occur if direction measurements were taken to the nearest 10 degrees). The grouped data was obtained by first generating a continuously distributed sample and then rounding each value.

We first of all explored type I error rate by generating samples from a uniform distribution, using the function *rcircularuniform* in *circular*. We set the nominal type I error rate at 0.05 and recorded the fraction of 10,000 uniform samples of a specified size that generated *p* values below 0.05.

For statistical power, we generated samples from non-uniform distributions and record the fraction of samples of a given size that produce *p* values below 0.05. We considered 12 different distributions including the von Mises (with concentration parameter *κ* = 1) and the skew normal (dispersion parameter = 1, skewness parameter = 30). Both of these were generated using the function *rcircmix* from the *R* package *NPCirc* (Oliveira Pérez et al. 2014). That same package was used to generate a symmetric (circular means at 0° and 180°) and asymmetric (0° and 120°) bimodal distribution made up of two von Mises distributions (*κ* = 5, equal proportions for both modes) and a symmetric (0°, 120° and 240°), as well as asymmetric (0°, 90° and 200°), trimodal one, made up of three von Mises distributions (with *κ* = 10, equal proportions for all three modes). In addition, we generated a cardioid distribution using the function *rcardioid* (*ρ* = 0.3), a Kato-Jones distribution using the function *rkatojones* (r = 0.7, *κ* = 2.3), a triangular distribution using the function *rtriangular* (ρ = 0.3), a wrapped Cauchy distribution using the function *rwrappedcauchy* (*ρ* = 0.7) and a wrapped stable distribution using the function *rwrappedstable* (scale = 1, index = 0.3, skewness = 1), all generated using the package *circular*. Finally, a wrapped normal distribution was generated using the function *rwrpnorm* from the package *CircStat* (Agostinelli and Lund 2018).

We also applied the original and TB versions of all tests to an example dataset explored by Tung and Jammalamadaka (2013). This consists of the directions taken by 13 released homing pigeons. The values are 20, 135, 145, 165, 170, 200, 300, 325, 335, 350, 350, 350 and 355 degrees from North. It appears these values have been rounded to the nearest 5 degrees. Further, by inspection, it looks like the data might be bimodally distributed, with values clustering around either North (0 or 360 degrees) or South (180 degrees).

## Results

**Fig. 1:**
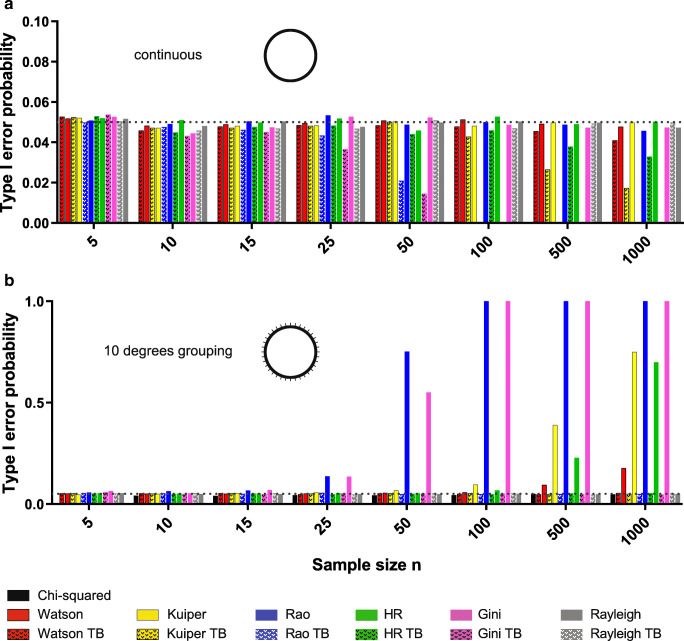
Type I error of the analysed tests on continuous and grouped uniform data. (**a**) The original Rayleigh, Rao, Gini, Kuiper, Watson and HR tests showed nominal type I error rates (5% indicated by the dashed line) when tested against continuous uniform distributions. However, the modified versions (TB, modified for a grouping of 10°) showed a decreasing type I error with an increasing sample size. (**b**) In contrast, when tested against a uniform distribution with grouped data (to the nearest 10°), the original test versions, with the exception of the Rayleigh and chi-squared tests, showed inflated type I error with increasing sample sizes, compared with the modified version control type I error rates

All six standard tests provided good control of type I error rate across all sample sizes explored when data were continuously distributed (Fig. 1a). The six TB tests produced type I error rates close to the nominal 5% level for the three smallest sample sizes (5, 10 and 15) but for higher samples of continuous data type I error rates were lower than expected for these tests. This effect occurs because these TB test versions all assume 36 bins even for continuous data, and therefore they are ‘correcting’ for bins where none exist. Of these, the HR test was the best behaved, producing values tolerably close to 5% for sample sizes of 25 and 50.

Behaviour was quite different when the original data was grouped (Fig. 1b). Now the six TB tests produced good control of type I error rates across all sample sizes. The standard Rao and Gini tests, however, had type I error rates close to the nominal level only for the three lowest sample sizes considered. After that they rose steeply, being close to twice the expected level for *n* = 25 and rising above 50% for *n* = 50. The type I error rate also climbed with sample size for the other standard tests with the notable exceptions of the Rayleigh and chi-squared tests. Indeed, the standard version of the Rayleigh test and the chi-squared test retains good control of type I error rate for all sample sizes considered. The underlying issue for most standard tests is that rounding can produce identical values, something that occurs with an increasing probability as sample size increases. Such identical values are treated by these tests as highly unlikely under the null hypothesis of uniformity.

**Fig. 2:**
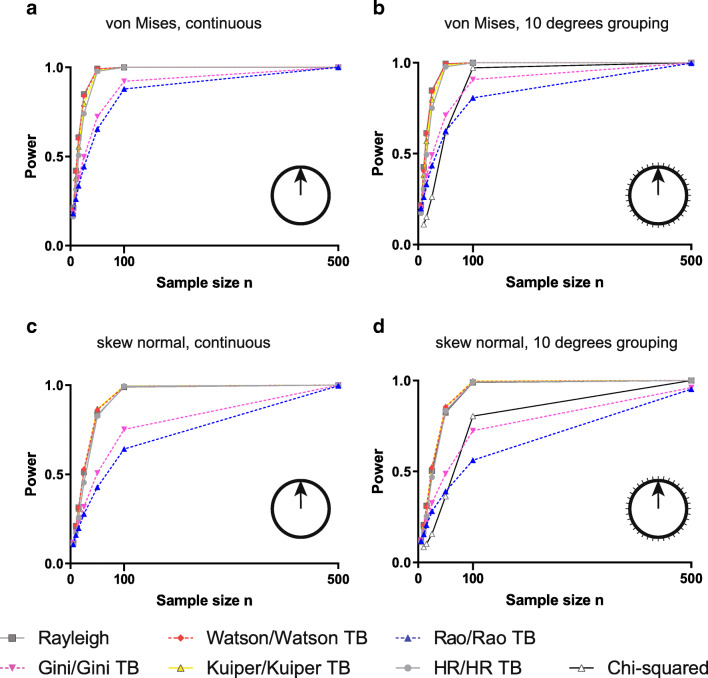
Power of the analysed tests on continuous and grouped unimodal data. Each of the original Rayleigh, Rao, Gini, Kuiper, Watson and HR tests (**a**) showed similar power levels compared to the modified version of the same test (**b**) when tested against continuous von Mises distributions, with the Rayleigh test being the most powerful test. Similarly, in the case of the skew normal distribution, the power levels of the original test versions (**c**) were comparable to the modified versions (**d**). However, in this case, both Rayleigh and HR tests gave almost identical results

Because of this separation in type I error rates, we compared the power performance within the group of six standard tests (applied to continuous data) and the separate group of five TB tests, Rayleigh test and the chi-squared test (applied to grouped data). For unimodal continuous data following a von Mises distribution, Rayleigh, Watson, Kuiper and HR tests offered broadly similar performance, with the Gini test being substantially less powerful but slightly more powerful than the Rao spacing test (Fig. 2a). Turning to grouped data for the same type of distribution, we saw a comparable relative performance of the TB tests—with Rayleigh, Watson, Kuiper and HR offering the best power, the chi-squared test offering a little less power, the Gini test being substantially less powerful and the Rao spacing test being a little less powerful again (Fig. 2b). We observed, in general, slightly lower power for grouped data using the TB tests (as would be expected, since the type I error rate is controlled for), but this reduction was relatively modest (particularly for the Watson, Kuiper and HR tests). The same qualitative trends were seen for data drawn from a skew normal distribution (Fig. 2c, d). Wrapped Cauchy and wrapped normal distributions revealed similar results, with the chi-squared test performing even worse, in the case of grouped data (Online Resource 1: Fig. A1 A-D). Interestingly, in the case of the wrapped stable distribution, the Rayleigh test was outperformed by Watson, Kuiper and HR tests and performed similarly to the chi-squared test on grouped data, while the Rao and Gini tests again performed poorly (Online Resource 1: Fig. A1 E, F). The cardioid and triangular distributions revealed almost identical results, which followed the general trend of the von Mises distribution. However, the power difference between the four tests with good performance (Rayleigh, Watson, Kuiper and HR test) and the less powerful tests (Rao, Gini and chi-squared test) was more pronounced for these distributions (Online Resource 1: Fig. A2 A-D). Interestingly, the Kato-Jones distribution showed low power for most tests, with HR, Watson and Kuiper tests outperforming the Rayleigh, Gini and Rao tests. In this distribution, the chi-squared test had slightly higher power than the Rayleigh test (Online Resource 1: Fig. A2 E, F).

**Fig. 3:**
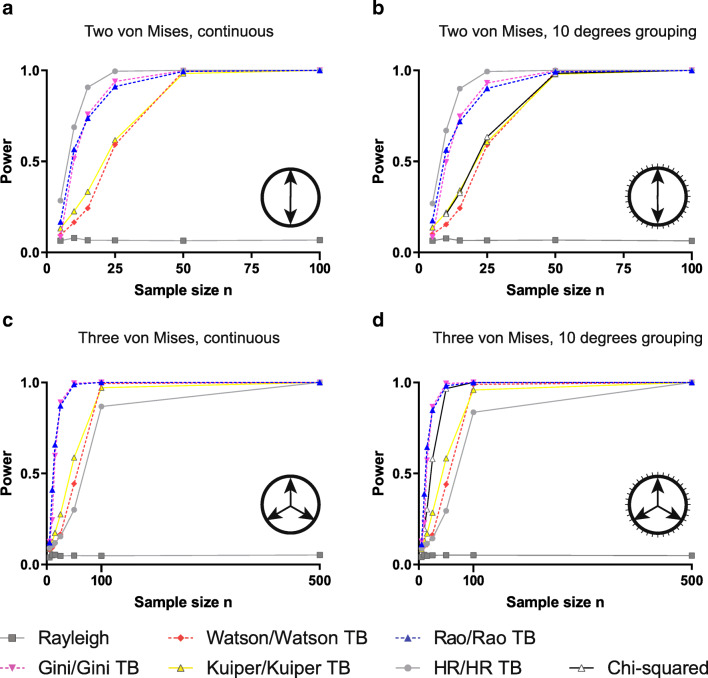
Power of the analysed tests on continuous and grouped multimodal data. Again, the original (**a**) and modified version (**b**) of the same test showed similar power, when tested against a bimodal von Mises distribution, with the HR test being the most powerful test. In the case of trimodal distributions, the power levels of the original test versions (**c**) also were comparable to the modified versions (**d**). However, in this case, the Rao and Gini tests outperformed the other alternative tests

Considering the relative performance of the six standard tests against a symmetric bimodal deviation, the Rayleigh test performed exceptionally poorly. The Rayleigh test is known to perform poorly for alternative distributions with mean resultant length equal to 0 (Mardia and Jupp 2000). This is because the Rayleigh test statistic is based on the mean resultant length of the data, and the mean resultant length for data from a symmetric multimodal distribution will be near 0, just as data from a uniform distribution would be. Hence, the Rayleigh test will not be able to discern between data from a uniform versus a symmetric multimodal distribution. Previous simulations by Landler et al. (2018) confirmed this. The other five tests had relatively similar performance with HR being generally best, followed by Gini and Rao, again having slightly lower power, and the Watson and Kuiper tests having substantially lower power (Fig. 3a). We saw essentially analogous performances from the corresponding four TB tests (Fig. 3b). There was only a very slight reduction in power as a result of grouping. Also the chi-squared test performed poorly, similarly to the Watson and Kuiper tests (Fig. 3b).

We also considered a trimodal deviation from uniformity for standard tests and continuous data and TB tests and grouped data, respectively. We found in both cases that the Rayleigh test offers no useful power. However, unlike the bimodal case, in the trimodal situation, the two methods based on spacings (Rao and Gini) comfortably outperformed the HR test, with the Rao spacing test very slightly outperforming the Gini test. The Watson and Kuiper tests were intermediate in their power between the Gini and HR tests. The chi-squared test showed good performance for this distribution in the case of grouped data, offering power intermediate between the Gini and Kuiper tests (Fig. 3c, d).

In the case of asymmetric bimodal distributions, test performances were similar amongst tests, with the exception of slightly reduced power of the Rayleigh and lower power of the chi-squared test, for grouped data (Online Resource 1: Fig. A3 A, B). The results changed when using an asymmetric trimodal distribution, the two most powerful tests were Gini and Rao, while all other tests show relatively poor performance, with the Rayleigh test being the least powerful (Online Resource 1: Fig. A3 C, D).

Finally, we tested a real-life example dataset of homing pigeon vanishing bearings explored by Tung and Jammalamadaka (2013). The application of the seven tests provided the following *p* values for standard (and, if available, TB) versions of the tests: Rayleigh 0.555, Watson *p* > 0.1 (0.138), Kuiper *p* > 0.15 (0.162), Rao 0.1 > *p* > 0.05 (0.0685), Gini 0.044 (0.048), HR 0.0034 (0.0039) and chi-squared 0.046. The anomalous performance of the Rayleigh test is not surprising given the apparent bimodal distribution of the data. The *p* value of the HR test was much below 0.05, the Gini and chi-squared tests were just below 0.05, the Rao spacing test was just slightly above 0.05 and the Watson and Kuiper tests considerably above 0.05—this is very much in line with the suggestion in Fig. 3 about the relative power of these tests to detect bimodal departures from uniformity. In this case, there was little difference between the *p* values suggested by the standard and TB versions of each test—likely because there was only one pair of tied values in the dataset.

## Discussion

Based on our simulations, we can refine previous guidance on testing the null hypothesis of uniformity with circular data. First, we consider continuous data. We note that in line with previous work (e.g. Landler et al. 2018), where expected deviations are unimodal, the Rayleigh test may be the most attractive option (although the Watson, Kuiper and HR tests can be used with only slight power loss). The HR test remains the most attractive option if the expected deviation is bimodal. For more complex deviations, the two tests based on spacings (Rao and Gini) might be more attractive. Of these, we would recommend the Rao spacing test (but only if the data is really continuous, see Landler et al. 2019). The Rao spacing test has the benefit of greater familiarity than the more recently proposed Gini test. Further, we could not find any circumstances where the Gini test offered substantially greater power.

Turning to grouped data, we would recommend that when data is grouped, researchers use the standard Rayleigh test or switch to a TB version of their test of choice as a means of preserving control of type I error rate without substantial loss of power. This switch is essential for spacing-based tests for all but the smallest sample sizes (for *N* > 20 as a rule of thumb). For the Watson, Kuiper and HR tests, it may be safe to use the standard tests for medium-sized samples, but there is no drawback to using the TB versions of these tests routinely for grouped data. Hence, we would recommend the routine use of TB versions of such tests, no matter the test or sample size, whenever data is grouped. We provide *R* code for these methods in the supplement of this article (Online Resource 2). Our recommendations for choice of (TB modified) test for grouped data mirrors our advice above on selecting a test for continuously distributed data. Finally, we cannot recommend using the chi-squared test, as it performed poorly in comparison to specific circular statistic tests. Further, the chi-squared test requires a minimal frequency of expected data points in each bin (recommended *n* ≥ 4, Batschelet 1981), a condition which is difficult to meet with small sample sizes, in particular when the data points are clustered.

Our analysis suggests that the issue of uncontrolled increase of type I error with aggregated data is common and widespread. We have highlighted the need for caution when applying aggregated data to statistical tests designed for continuously distributed circular data in the context of testing a single sample for uniformity, the most commonly applied statistical procedure on circular data. Pending further research, we would recommend that the method suggested here—tie-breaking through addition of small perturbations followed by evaluation by simulation—should be effective in any statistical procedure involving aggregated circular data.

## Electronic supplementary material

ESM 1 (PDF 1018 kb)

ESM 2 (PDF 158 kb)

## References

[CR1] Agostinelli C, Lund U (2017) R package circular: Circular Statistics (version 0.4-93). https://r-forge.r-project.org/projects/circular/

[CR2] Agostinelli C, Lund U (2018) R package CircStats: Circular Statistics (version 0.2-6). https://cran.r-project.org/web/packages/CircStats/

[CR3] Batschelet E (1981). Circular statistics in biology.

[CR4] Dwass M (1957). Modified randomization tests for nonparametric hypotheses. Ann Math Stat.

[CR5] Fisher NI (1995). Statistical analysis of circular data.

[CR6] Fitak RR, Johnsen S (2017). Bringing the analysis of animal orientation data full circle: model-based approaches with maximum likelihood. J Exp Biol.

[CR7] Freedman LS (1979). The use of a Kolmogorov-Smirnov type statistic in testing hypotheses about seasonal variation. J Epidemiol Commun Health.

[CR8] Hermans M, Rasson J (1985). A new Sobolev test for uniformity on the circle. Biometrika.

[CR9] Humphreys RK, Ruxton GD (2017). Consequences of grouped data for testing for departure from circular uniformity. Behav Ecol Sociobiol.

[CR10] Jammalamadaka SR, SenGupta A (2001). Topics in circular statistics.

[CR11] Landler L, Ruxton GD, Malkemper EP (2018). Circular data in biology: advice for effectively implementing statistical procedures. Behav Ecol Sociobiol.

[CR12] Landler L, Ruxton GD, Malkemper EP (2019). Circular statistics meets practical limitations: a simulation-based Rao’s spacing test for non-continuous data. Mov Ecol.

[CR13] Ley C, Verdebout T (2017). Modern directional statistics.

[CR14] Mardia KV, Jupp PE (2000). Directional statistics.

[CR15] Oliveira Pérez M, Crujeiras Casais RM, Rodríguez Casal A (2014). NPCirc: an R package for nonparametric circular methods. J Stat Soft.

[CR16] Pewsey A, Neuhäuser M, Ruxton GD (2013). Circular statistics in R.

[CR17] Pycke JR (2010). Some tests for uniformity of circular distributions powerful against multimodal alternatives. Can J Stat.

[CR18] Ruxton GD, Neuhäuser M (2010). Good practice in testing for an association in contingency tables. Behav Ecol Sociobiol.

[CR19] Taube JS (2007). The head direction signal: origins and sensory-motor integration. Annu Rev Neurosci.

[CR20] Tung DD, Jammalamadaka SR (2013). On the Gini mean difference test for circular data. Commun Stat Theory Methods.

