# Supplementary material for: Grouped circular data in biology: advice for effectively implementing statistical procedures
Source: Behav Ecol Sociobiol. 2020 Jul 20;74(8):100. doi: 10.1007/s00265-020-02881-6 (PMC7373216; doi:10.1007/s00265-020-02881-6)
Supplement: Supplementary file 1 — (PDF 1018 kb) [file 265_2020_2881_MOESM1_ESM.pdf]

Online Resource 1: Supplementary figures

Behavioural Ecology and Sociobiology

Grouped circular data in biology: advice for effectively implementing statistical procedures

Lukas Landler[1], Graeme D. Ruxton[2], E. Pascal Malkemper[3]

Affiliations

[1] Institute of Zoology, University of Natural Resources and Life Sciences, Gregor-Mendel-Straße 33/I, 1180 Vienna, Austria

[2] School of Biology, University of St Andrews, St Andrews KY16 9TH, UK

[3] Max Planck Research Group Neurobiology of Magnetoreception, Center of Advanced European Studies and Research (caesar), Ludwig-Erhard-Allee 2, Bonn 53175, Germany

Corresponding author email address: pascal.malkemper@caesar.de

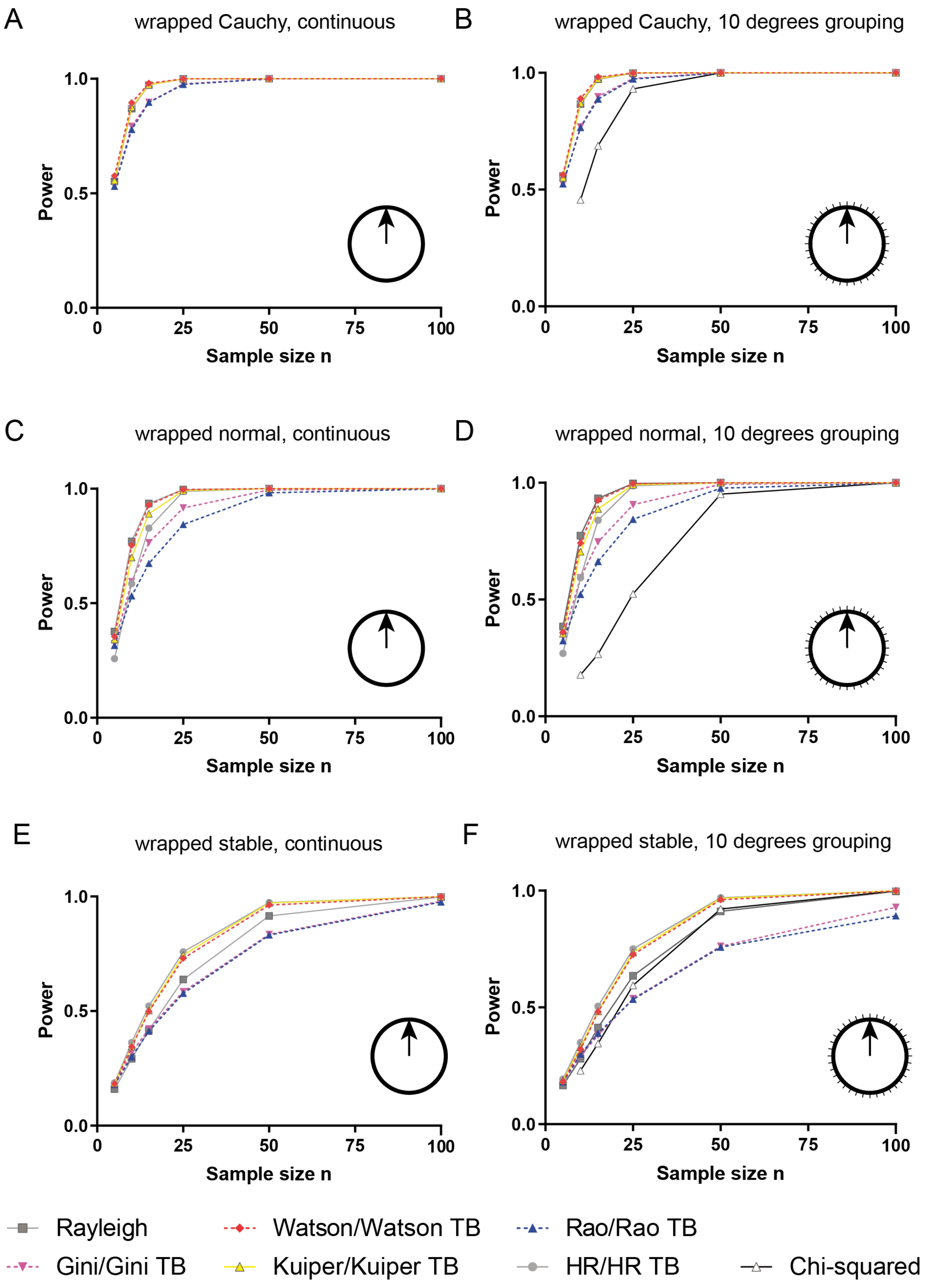
A
wrapped Cauchy, continuous
B
wrapped Cauchy, 10 degrees grouping
C
wrapped normal, continuous
D
wrapped normal, 10 degrees grouping
E
wrapped stable, continuous
F
wrapped stable, 10 degrees grouping
Power
Sample size n
0.0
0.5
1.0
0
25
50
75
100
Rayleigh
Watson/Watson TB
Rao/Rao TB
Gini/Gini TB
Kuiper/Kuiper TB
HR/HR TB
Chi-squared

Fig. A1 Power of the analysed tests on continuous and grouped unimodal data from different distributions. Each of the original Rayleigh, Rao, Gini, Kuiper, Watson and HR tests (A) showed similar power levels compared to the modified version of the same test (B) when tested against continuous wrapped Cauchy distributions, with the Gini and Rao tests having the lowest power. Similarly, in the case of wrapped normal distributions the power levels of the original test versions (C) were comparable to the modified versions (D). The same was true for a wrapped stable distribution (E, F). However, in this case the power was relatively low for all tests with the HR, Kuiper and Watson tests performing best. The chi-squared test offered lower power than most other tests

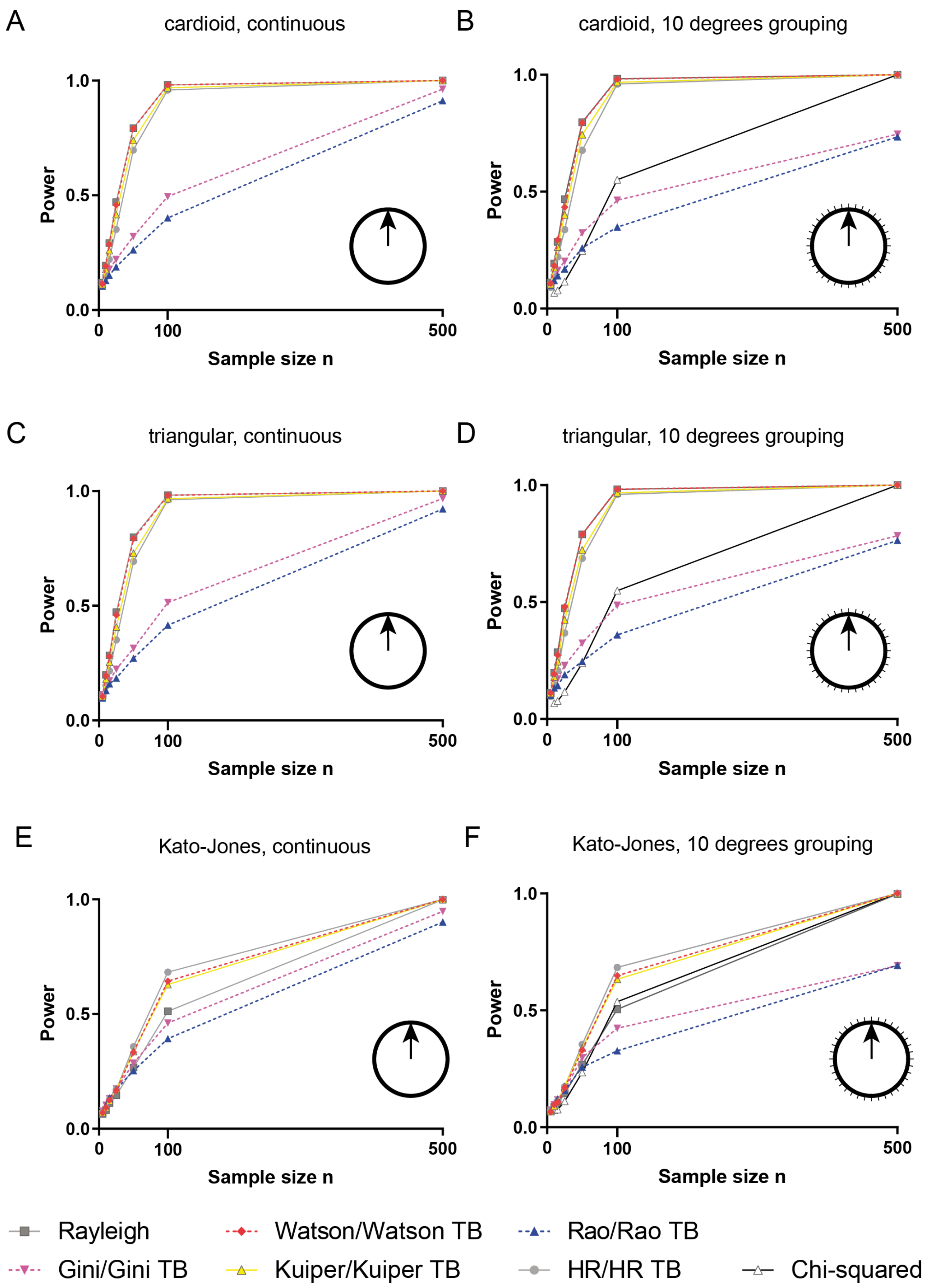
A
cardioid, continuous
B
cardioid, 10 degrees grouping
C
triangular, continuous
D
triangular, 10 degrees grouping
E
Kato-Jones, continuous
F
Kato-Jones, 10 degrees grouping
Power
Sample size n
0.0
0.5
1.0
0
100
500
Rayleigh
Watson/Watson TB
Rao/Rao TB
Gini/Gini TB
Kuiper/Kuiper TB
HR/HR TB
Chi-squared

Fig. A2 Power of the analysed tests on continuous and grouped unimodal data from different distributions. Each of the original Rayleigh, Rao, Gini, Kuiper, Watson and HR tests (A) showed similar power levels compared to the modified version of the same test (B) when tested against continuous cardioid distributions, with the Rayleigh and Watson tests giving the best results. Similarly, in the case of triangular distributions the power levels of the original test versions (C) were comparable to the modified versions (D). The same was true for a Kato-Jones distribution (E, F). However, in this case the power was relatively low for all tests with the HR test performing best. In all grouped situations, the chi-squared test gave only average power

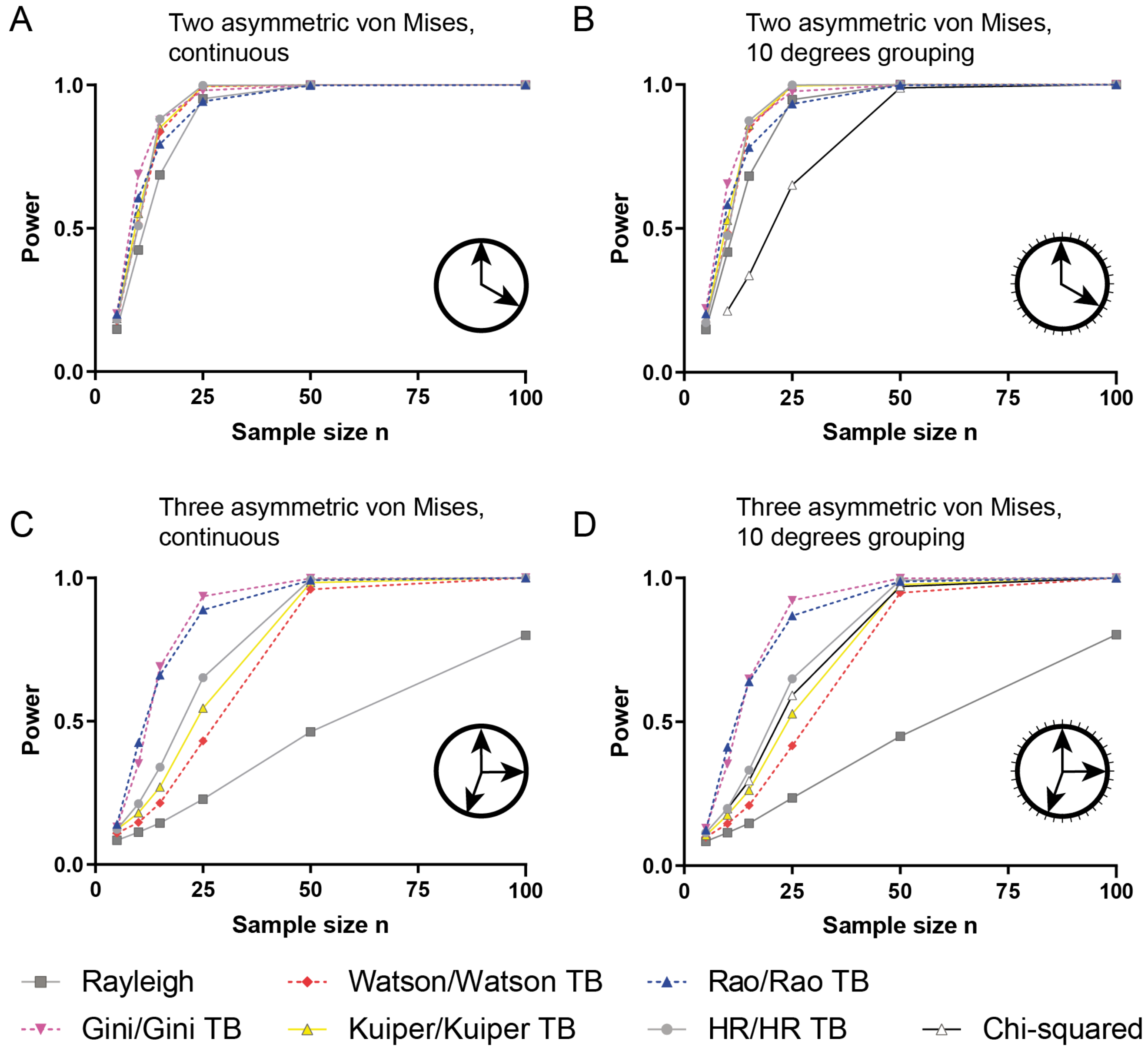

Fig. A3 Power of the analysed tests on continuous and grouped asymmetric multimodal data. Again, original (A) and modified version (B) of the same test showed similar power, when tested against a bimodal von Mises distribution, with the HR tests being the most powerful and the chi-squared the least powerful test. In the case of asymmetric trimodal distributions the power levels of the original test versions (C) were comparable to the modified versions (D). Here, the Rao and Gini test outperformed the other alternative tests while the Rayleigh test had the lowest power
