# Supplementary material for: Grouped circular data in biology: advice for effectively implementing statistical procedures
Source: Behav Ecol Sociobiol. 2020 Jul 20;74(8):100. doi: 10.1007/s00265-020-02881-6 (PMC7373216; doi:10.1007/s00265-020-02881-6)
Supplement: Supplementary file 2 — (PDF 158 kb) [file 265_2020_2881_MOESM2_ESM.pdf]

Online Resource 2: R code

Behavioural Ecology and Sociobiology

Grouped circular data in biology: advice for effectively implementing statistical procedures

Lukas Landler[1], Graeme D. Ruxton[2], E. Pascal Malkemper[3]

Affiliations

[1] Institute of Zoology, University of Natural Resources and Life Sciences, Gregor-Mendel-Straße 33/I, 1180 Vienna, Austria

[2] School of Biology, University of St Andrews, St Andrews KY16 9TH, UK

[3] Max Planck Research Group Neurobiology of Magnetoreception, Center of Advanced European Studies and Research (caesar), Ludwig-Erhard-Allee 2, Bonn 53175, Germany

Corresponding author email address: pascal.malkemper@caesar.de

1. **R code for the self-written functions and an example of applying the tests to a circular data set - (input data in radians!!! – see page 10 for input in degrees)**

```
#the critical value functions need to be run in order for the p-value functions to work!

#loading library
library(circular)

#setting function for Gini test
#critical value calculation
GiniTestValue <- function(sample){n <- length(sample)
        f <- sort(sample)
        fplus <- c(f[2:n],f[1])
        D <- fplus - f
        D[n] <- (2*pi) - f[n] + f[1]
        nD <- n*D
        Gn <- 0
        for (i in 1:(n-1)){
        for (j in (i+1):n){Gn <- Gn + (0.5*abs(nD[i] - nD[j]))}}
        Gn <- 2*Gn/(n*(n-1))
        return(Gn)}
#p value calculation for grouped data (m is the number of bins and k is the kappa for the error distribution
GiniTestGroupRad <- function(sample, m, k){
  sample<-circular(sample)
  sample<- ifelse((sample>(2*pi)),(sample-(2*pi)), sample)
  sample<- ifelse((sample<(0)),(sample+(2*pi)), sample)
  sample<- ifelse((sample>(2*pi)),(sample-(2*pi)), sample)
  sample<- ifelse((sample<(0)),(sample+(2*pi)), sample)
  n <- length(sample)
  univals <- 9999
  testset<- rep(0,univals)
  for (f in 1:univals){
    data1 <- rcircularuniform(n, control.circular=list(units="radians"))
    data1 <- trunc(data1*m/(2*pi))
```

```
    data1 <- data1*2*pi/m
    errorsamp <- rvonmises(n, 0, k,control.circular=list(units="radians"))
    data1 <- data1+errorsamp
    data1 <- ifelse((data1>(2*pi)),(data1-(2*pi)), data1)
    testset[f] <- GiniTestValue(data1)}
  errorsamp2 <- rvonmises(n, 0, k,control.circular=list(units="radians"))
   sample<-sample+errorsamp2
   sample<- ifelse((sample>(2*pi)),(sample-(2*pi)), sample)
  Tsample <- GiniTestValue(sample)
  counter <- 0
  for(j in 1:univals){if(testset[j]>=Tsample){counter <- counter+1}}
  p <- counter/(univals+1)
  return(p)}
```

#p value calculation for continuous data (m is the number of bins and k is the kappa for the error distribution

```
GiniTestUngroupedRad <- function(sample){
  sample<-circular(sample)
  sample<- ifelse((sample>(2*pi)),(sample-(2*pi)), sample)
  sample<- ifelse((sample<(0)),(sample+(2*pi)), sample)
  sample<- ifelse((sample>(2*pi)),(sample-(2*pi)), sample)
  sample<- ifelse((sample<(0)),(sample+(2*pi)), sample)
  n <- length(sample)
  univals <- 999
  testset<- rep(0,univals)
  for (f in 1:univals){
    data1 <- rcircularuniform(n, control.circular=list(units="radians"))
    testset[f] <- GiniTestValue(data1)}
 Tsample <- GiniTestValue(sample)
  counter <- 0
  for(j in 1:univals){if(testset[j]>=Tsample){counter <- counter+1}}
  p <- counter/(univals+1)
  return(p)}
```

#setting function for HR

#critical value calculation

```
HermansRasson2T <- function(sample){
```

```
            n <- length(sample)
            total <- 0
            for (i in 1:n){
                    for (j in 1:n){ total <- total + abs(abs(sample[i]-sample[j])-pi)-
(pi/2)
        total <- total - (2.895*(abs(sin(sample[i]-sample[j]))-(2/pi)))}}
            T <- total/n
            return(T)}
```

#p value calculation for grouped data (m is the number of bins and k is the kappa for the error distribution

```
HermansRasson2PGroupedRad <- function(sample, m, k){
  sample<-circular(sample)
  sample<- ifelse((sample>(2*pi)),(sample-(2*pi)), sample)
  sample<- ifelse((sample<(0)),(sample+(2*pi)), sample)
  sample<- ifelse((sample>(2*pi)),(sample-(2*pi)), sample)
  sample<- ifelse((sample<(0)),(sample+(2*pi)), sample)
  n <- length(sample)
  univals <- 9999
  testset<- rep(0,univals)
  for (f in 1:univals){
    data1 <- rcircularuniform(n, control.circular=list(units="radians"))
    data1 <- trunc(data1*m/(2*pi))
    data1 <- data1*2*pi/m
    errorsamp <- rvonmises(n, 0, k,control.circular=list(units="radians"))
    data1 <- data1+errorsamp
    data1 <- ifelse((data1>(2*pi)),(data1-(2*pi)), data1)
    testset[f] <- HermansRasson2T(data1)}
  errorsamp2 <- rvonmises(n, 0, k,control.circular=list(units="radians"))
   sample<-sample+errorsamp2
   sample<- ifelse((sample>(2*pi)),(sample-(2*pi)), sample)
  Tsample <- HermansRasson2T(sample)
  counter <- 0
  for(j in 1:univals){if(testset[j]>=Tsample){counter <- counter+1}}
  p <- counter/(univals+1)
  return(p)}
```

#p value calculation for continuous data (m is the number of bins and k is the kappa for the error distribution

```
HermansRasson2PRad<- function(sample){
                sample<-circular(sample)
                sample<- ifelse((sample>(2*pi)),(sample-(2*pi)), sample)
                sample<- ifelse((sample<(0)),(sample+(2*pi)), sample)
                sample<- ifelse((sample>(2*pi)),(sample-(2*pi)), sample)
                sample<- ifelse((sample<(0)),(sample+(2*pi)), sample)
                univals <- 9999
                n <- length(sample)
                sample<-circular(sample, units ="radians")
                testset<- rep(0,univals)
                for (f in 1:univals){
                        data1 <- matrix(rcircularuniform(n,
control.circular=list(units="radians")))
                        testset[f] <- HermansRasson2T(data1)}
                Tsample <- HermansRasson2T(sample)
                counter <- 1
                for(j in 1:univals){if(testset[j]>=Tsample){counter <- counter+1}}
                p <- counter/(univals+1)
                return(p)}
```

#setting function for Rao's spacing test

#critical value calculation

```
RaoTestValue <- function(sample){n <- length(sample)
f <- sort(sample)
fplus <- c(f[2:n],f[1])
T <- fplus - f
T[n] <- (2*pi) - f[n] + f[1]
abs_diff <- abs(T-(2*pi/n))
U <- 0.5*sum(abs_diff)
return(U)}
```

#p value calculation for grouped data (m is the number of bins and k is the kappa for the error distribution

```
RaoPGroupedRad <- function(sample, m, k){
  sample<-circular(sample)
```

```
  sample<- ifelse((sample>(2*pi)),(sample-(2*pi)), sample)
  sample<- ifelse((sample<(0)),(sample+(2*pi)), sample)
  sample<- ifelse((sample>(2*pi)),(sample-(2*pi)), sample)
  sample<- ifelse((sample<(0)),(sample+(2*pi)), sample)
  n <- length(sample)
  univals <- 9999
  testset<- rep(0,univals)
  for (f in 1:univals){
    data1 <- rcircularuniform(n, control.circular=list(units="radians"))
    data1 <- trunc(data1*m/(2*pi))
    data1 <- data1*2*pi/m
    errorsamp <- rvonmises(n, 0, k,control.circular=list(units="radians"))
    data1 <- data1+errorsamp
    data1 <- ifelse((data1>(2*pi)),(data1-(2*pi)), data1)
    testset[f] <- RaoTestValue(data1)}
  errorsamp2 <- rvonmises(n, 0, k,control.circular=list(units="radians"))
   sample<-sample+errorsamp2
   sample<- ifelse((sample>(2*pi)),(sample-(2*pi)), sample)
  Tsample <- RaoTestValue(sample)
  counter <- 0
  for(j in 1:univals){if(testset[j]>=Tsample){counter <- counter+1}}
  p <- counter/(univals+1)
  return(p)}
```

#Rao's spacing test function used in this study for continuous data (uncorrected for grouping – from package "circular") – example can be run after running the sample test code underneath

```
rao.spacing.test(sampletest,alpha=0.05)
```

#Watson test function (critical value is calculated using the function watson.test() from the package circular

#p value calculation for grouped data (m is the number of bins and k is the kappa for the error distribution

```
WatsonPGroupedRad <- function(sample, m, k){
  sample<-circular(sample)
```

```
  sample<- ifelse((sample>(2*pi)),(sample-(2*pi)), sample)
  sample<- ifelse((sample<(0)),(sample+(2*pi)), sample)
  sample<- ifelse((sample>(2*pi)),(sample-(2*pi)), sample)
  sample<- ifelse((sample<(0)),(sample+(2*pi)), sample)
  n <- length(sample)
  univals <- 9999
  testset<- rep(0,univals)
  for (f in 1:univals){
    data1 <- rcircularuniform(n, control.circular=list(units="radians"))
    data1 <- trunc(data1*m/(2*pi))
    data1 <- data1*2*pi/m
    errorsamp <- rvonmises(n, 0, k,control.circular=list(units="radians"))
    data1 <- data1+errorsamp
    data1 <- ifelse((data1>(2*pi)),(data1-(2*pi)), data1)
    watson1 <-watson.test(data1)
    testset[f] <-watson1$statistic }
  errorsamp2 <- rvonmises(n, 0, k,control.circular=list(units="radians"))
  sample<-sample+errorsamp2
  sample<- ifelse((sample>(2*pi)),(sample-(2*pi)), sample)
  watson2 <-watson.test(sample)
  Tsample <- watson2$statistic
  counter <- 0
  for(j in 1:univals){if(testset[j]>=Tsample){counter <- counter+1}}
  p <- counter/(univals+1)
  return(p)}
```

#Kuiper test function (critical value is calculated using the function kuiper.test() from the package circular

#p value calculation for grouped data (m is the number of bins and k is the kappa for the error distribution

```
KuiperPGroupedRad <- function(sample, m, k){
  sample<-circular(sample)
  sample<- ifelse((sample>(2*pi)),(sample-(2*pi)), sample)
  sample<- ifelse((sample<(0)),(sample+(2*pi)), sample)
  sample<- ifelse((sample>(2*pi)),(sample-(2*pi)), sample)
  sample<- ifelse((sample<(0)),(sample+(2*pi)), sample)
  n <- length(sample)
```

```
  univals <- 9999
  testset<- rep(0,univals)
  for (f in 1:univals){
    data1 <- rcircularuniform(n, control.circular=list(units="radians"))
    data1 <- trunc(data1*m/(2*pi))
    data1 <- data1*2*pi/m
    errorsamp <- rvonmises(n, 0, k,control.circular=list(units="radians"))
    data1 <- data1+errorsamp
    data1 <- ifelse((data1>(2*pi)),(data1-(2*pi)), data1)
    kuiper1 <-kuiper.test(data1)
    testset[f] <-kuiper1$statistic }
  errorsamp2 <- rvonmises(n, 0, k,control.circular=list(units="radians"))
  sample<-sample+errorsamp2
  sample<- ifelse((sample>(2*pi)),(sample-(2*pi)), sample)
  kuiper2 <-kuiper.test(sample)
  Tsample <- kuiper2$statistic
  counter <- 0
  for(j in 1:univals){if(testset[j]>=Tsample){counter <- counter+1}}
  p <- counter/(univals+1)
  return(p)}
```

#Rayleigh test function (from package "circular") – this example can be run after running the sample test code underneath

```
rayleigh.test(circular(sampletest))
```

# Preparing the test sample 'pigeon data'

```
sampletest <- rad(c(20,135,145,165,170,200,300,325,335,350,350,350,355))
```

#Applying the Gini test

```
GiniTestGroupRad(sampletest, 72,1000)
GiniTestUngroupedRad (sampletest)
```

#applying the HR test

```
HermansRasson2PGroupedRad(sampletest, 72,1000)
HermansRasson2PRad(sampletest)
```

#applying the Rao's spacing test

RaoPGroupedRad(sampletest,72,1000)

rao.spacing.test(sampletest)

#Applying the rayleigh test

rayleigh.test(sampletest)

#Applying the Watson test

WatsonPGroupedRad(sampletest, 72,1000)

watson.test(sampletest)

#Applying the Kuiper test

KuiperPGroupedRad(sampletest, 72,1000)

kuiper.test(sampletest)

2. **R code for the self-written functions and an example of applying the tests to a circular data set – (input data in degrees!!!)**

#the critical value functions need to be run in order for the p-value functions to work!

#loading library

```
library(circular)
```

#setting function for Gini test

#critical value calculation

```
GiniTestValue <- function(sample){n <- length(sample)
        f <- sort(sample)
        fplus <- c(f[2:n],f[1])
        D <- fplus - f
        D[n] <- (2*pi) - f[n] + f[1]
        nD <- n*D
        Gn <- 0
        for (i in 1:(n-1)){
        for (j in (i+1):n){Gn <- Gn + (0.5*abs(nD[i] - nD[j]))}}
        Gn <- 2*Gn/(n*(n-1))
        return(Gn)}
```

#p value calculation for grouped data (m is the number of bins and k is the kappa for the error distribution

```
GiniTestGroupDeg <- function(sample, m, k){
  sample<-circular(sample)
  sample<-rad(sample)
  sample<- ifelse((sample>(2*pi)),(sample-(2*pi)), sample)
  sample<- ifelse((sample<(0)),(sample+(2*pi)), sample)
  sample<- ifelse((sample>(2*pi)),(sample-(2*pi)), sample)
  sample<- ifelse((sample<(0)),(sample+(2*pi)), sample)
  n <- length(sample)
  univals <- 9999
  testset<- rep(0,univals)
  for (f in 1:univals){
    data1 <- rcircularuniform(n, control.circular=list(units="radians"))
    data1 <- trunc(data1*m/(2*pi))
```

```
    data1 <- data1*2*pi/m
    errorsamp <- rvonmises(n, 0, k,control.circular=list(units="radians"))
    data1 <- data1+errorsamp
    data1 <- ifelse((data1>(2*pi)),(data1-(2*pi)), data1)
    testset[f] <- GiniTestValue(data1)}
  errorsamp2 <- rvonmises(n, 0, k,control.circular=list(units="radians"))
   sample<-sample+errorsamp2
   sample<- ifelse((sample>(2*pi)),(sample-(2*pi)), sample)
  Tsample <- GiniTestValue(sample)
  counter <- 0
  for(j in 1:univals){if(testset[j]>=Tsample){counter <- counter+1}}
  p <- counter/(univals+1)
  return(p)}
```

#p value calculation for continuous data (m is the number of bins and k is the kappa for the error distribution

```
GiniTestUngroupedDeg <- function(sample){
  sample<-circular(sample)
  sample<-rad(sample)
  sample<- ifelse((sample>(2*pi)),(sample-(2*pi)), sample)
  sample<- ifelse((sample<(0)),(sample+(2*pi)), sample)
  sample<- ifelse((sample>(2*pi)),(sample-(2*pi)), sample)
  sample<- ifelse((sample<(0)),(sample+(2*pi)), sample)
  n <- length(sample)
  univals <- 999
  testset<- rep(0,univals)
  for (f in 1:univals){
    data1 <- rcircularuniform(n, control.circular=list(units="radians"))
    testset[f] <- GiniTestValue(data1)}
 Tsample <- GiniTestValue(sample)
  counter <- 0
  for(j in 1:univals){if(testset[j]>=Tsample){counter <- counter+1}}
  p <- counter/(univals+1)
  return(p)}
```

#setting function for HR

#critical value calculation

```
HermansRasson2T <- function(sample){
                    n <- length(sample)
                    total <- 0
                    for (i in 1:n){
                              for (j in 1:n){ total <- total + abs(abs(sample[i]-sample[j])-pi)-
(pi/2)
          total <- total - (2.895*(abs(sin(sample[i]-sample[j]))-(2/pi)))}}
                    T <- total/n
                    return(T)}
```

#p value calculation for grouped data (m is the number of bins and k is the kappa for the error distribution)

```
HermansRasson2PGroupedDeg <- function(sample, m, k){
  sample<-circular(sample)
  sample<-rad(sample)
  sample<- ifelse((sample>(2*pi)),(sample-(2*pi)), sample)
  sample<- ifelse((sample<(0)),(sample+(2*pi)), sample)
  sample<- ifelse((sample>(2*pi)),(sample-(2*pi)), sample)
  sample<- ifelse((sample<(0)),(sample+(2*pi)), sample)
  n <- length(sample)
  univals <- 9999
  testset<- rep(0,univals)
  for (f in 1:univals){
    data1 <- rcircularuniform(n, control.circular=list(units="radians"))
    data1 <- trunc(data1*m/(2*pi))
    data1 <- data1*2*pi/m
    errorsamp <- rvonmises(n, 0, k,control.circular=list(units="radians"))
    data1 <- data1+errorsamp
    data1 <- ifelse((data1>(2*pi)),(data1-(2*pi)), data1)
    testset[f] <- HermansRasson2T(data1)}
  errorsamp2 <- rvonmises(n, 0, k,control.circular=list(units="radians"))
   sample<-sample+errorsamp2
   sample<- ifelse((sample>(2*pi)),(sample-(2*pi)), sample)
  Tsample <- HermansRasson2T(sample)
  counter <- 0
  for(j in 1:univals){if(testset[j]>=Tsample){counter <- counter+1}}
  p <- counter/(univals+1)
  return(p)}
```

#p value calculation for continuous data (m is the number of bins and k is the kappa for the error distribution

```
HermansRasson2PDeg<- function(sample){
                sample<-circular(sample)
                sample <- rad(sample)
                sample<- ifelse((sample>(2*pi)),(sample-(2*pi)), sample)
                sample<- ifelse((sample<(0)),(sample+(2*pi)), sample)
                sample<- ifelse((sample>(2*pi)),(sample-(2*pi)), sample)
                sample<- ifelse((sample<(0)),(sample+(2*pi)), sample)
                univals <- 9999
                n <- length(sample)
                sample<-circular(sample, units ="radians")
                testset<- rep(0,univals)
                for (f in 1:univals){
                        data1           <-          matrix(rcircularuniform(n,
control.circular=list(units="radians")))
                        testset[f] <- HermansRasson2T(data1)}
                Tsample <- HermansRasson2T(sample)
                counter <- 1
                for(j in 1:univals){if(testset[j]>=Tsample){counter <- counter+1}}
                p <- counter/(univals+1)
                return(p)}
```

#setting function for Rao's spacing test
#critical value calculation

```
RaoTestValue <- function(sample){n <- length(sample)
f <- sort(sample)
fplus <- c(f[2:n],f[1])
T <- fplus - f
T[n] <- (2*pi) - f[n] + f[1]
abs_diff <- abs(T-(2*pi/n))
U <- 0.5*sum(abs_diff)
return(U)}
```

#p value calculation for grouped data (m is the number of bins and k is the kappa for the error distribution

```
RaoPGroupedDeg <- function(sample, m, k){
  sample<-circular(sample)
  sample <- rad(sample)
  sample<- ifelse((sample>(2*pi)),(sample-(2*pi)), sample)
  sample<- ifelse((sample<(0)),(sample+(2*pi)), sample)
  sample<- ifelse((sample>(2*pi)),(sample-(2*pi)), sample)
  sample<- ifelse((sample<(0)),(sample+(2*pi)), sample)
  n <- length(sample)
  univals <- 9999
  testset<- rep(0,univals)
  for (f in 1:univals){
    data1 <- rcircularuniform(n, control.circular=list(units="radians"))
    data1 <- trunc(data1*m/(2*pi))
    data1 <- data1*2*pi/m
    errorsamp <- rvonmises(n, 0, k,control.circular=list(units="radians"))
    data1 <- data1+errorsamp
    data1 <- ifelse((data1>(2*pi)),(data1-(2*pi)), data1)
    testset[f] <- RaoTestValue(data1)}
  errorsamp2 <- rvonmises(n, 0, k,control.circular=list(units="radians"))
   sample<-sample+errorsamp2
   sample<- ifelse((sample>(2*pi)),(sample-(2*pi)), sample)
  Tsample <- RaoTestValue(sample)
  counter <- 0
  for(j in 1:univals){if(testset[j]>=Tsample){counter <- counter+1}}
  p <- counter/(univals+1)
  return(p)}
```

#Rao's spacing test function used in this study for continuous data (uncorrected for grouping – from package “circular”) – example can be run after running the sample test code underneath

```
rao.spacing.test(circular(sampletest, units ="degrees"),alpha=0.05)
```

#Watson test function (critical value is calculated using the function watson.test() from the package circular

#p value calculation for grouped data (m is the number of bins and k is the kappa for the error distribution

```
WatsonPGroupedDeg <- function(sample, m, k){
```

```
  sample<-circular(sample)
  sample<-rad(sample)
  sample<- ifelse((sample>(2*pi)),(sample-(2*pi)), sample)
  sample<- ifelse((sample<(0)),(sample+(2*pi)), sample)
  sample<- ifelse((sample>(2*pi)),(sample-(2*pi)), sample)
  sample<- ifelse((sample<(0)),(sample+(2*pi)), sample)
  n <- length(sample)
  univals <- 9999
  testset<- rep(0,univals)
  for (f in 1:univals){
    data1 <- rcircularuniform(n, control.circular=list(units="radians"))
    data1 <- trunc(data1*m/(2*pi))
    data1 <- data1*2*pi/m
    errorsamp <- rvonmises(n, 0, k,control.circular=list(units="radians"))
    data1 <- data1+errorsamp
    data1 <- ifelse((data1>(2*pi)),(data1-(2*pi)), data1)
    watson1 <-watson.test(data1)
    testset[f] <-watson1$statistic }
  errorsamp2 <- rvonmises(n, 0, k,control.circular=list(units="radians"))
  sample<-sample+errorsamp2
  sample<- ifelse((sample>(2*pi)),(sample-(2*pi)), sample)
  watson2 <-watson.test(sample)
  Tsample <- watson2$statistic
  counter <- 0
  for(j in 1:univals){if(testset[j]>=Tsample){counter <- counter+1}}
  p <- counter/(univals+1)
  return(p)}
```

#Kuiper test function (critical value is calculated using the function kuiper.test() from the package circular

#p value calculation for grouped data (m is the number of bins and k is the kappa for the error distribution

```
KuiperPGroupedDeg <- function(sample, m, k){
  sample<-circular(sample)
  sample<-rad(sample)
  sample<- ifelse((sample>(2*pi)),(sample-(2*pi)), sample)
  sample<- ifelse((sample<(0)),(sample+(2*pi)), sample)
```

```
  sample<- ifelse((sample>(2*pi)),(sample-(2*pi)), sample)
  sample<- ifelse((sample<(0)),(sample+(2*pi)), sample)
  n <- length(sample)
  univals <- 9999
  testset<- rep(0,univals)
  for (f in 1:univals){
    data1 <- rcircularuniform(n, control.circular=list(units="radians"))
    data1 <- trunc(data1*m/(2*pi))
    data1 <- data1*2*pi/m
    errorsamp <- rvonmises(n, 0, k,control.circular=list(units="radians"))
    data1 <- data1+errorsamp
    data1 <- ifelse((data1>(2*pi)),(data1-(2*pi)), data1)
    kuiper1 <-kuiper.test(data1)
    testset[f] <-kuiper1$statistic }
  errorsamp2 <- rvonmises(n, 0, k,control.circular=list(units="radians"))
  sample<-sample+errorsamp2
  sample<- ifelse((sample>(2*pi)),(sample-(2*pi)), sample)
  kuiper2 <-kuiper.test(sample)
  Tsample <- kuiper2$statistic
  counter <- 0
  for(j in 1:univals){if(testset[j]>=Tsample){counter <- counter+1}}
  p <- counter/(univals+1)
  return(p)}
```

#Rayleigh test function (from package “circular”) – example can be run after running the sample test code underneath

```
rayleigh.test(circular(sampletest, units ="degrees"))
```

#Preparing the test sample ‘pigeon data’

```
sampletest <- c(20,135,145,165,170,200,300,325,335,350,350,350,355)
```

#Applying the Gini test

```
GiniTestGroupDeg(sampletest, 72,1000)
GiniTestUngroupedDeg(sampletest)
```

#applying the HR test

```
HermansRasson2PGroupedDeg(sampletest, 72,1000)
```

HermansRasson2PDeg(sampletest)

#applying the Rao's spacing test

RaoPGroupedDeg(sampletest, 72,1000)

rao.spacing.test(circular(sampletest, units ="degrees"),alpha=0.05)

#Applying the rayleigh test

rayleigh.test(circular(sampletest, units ="degrees"))

#Applying the Watson test

WatsonPGroupedDeg(sampletest, 72,1000)

watson.test(sampletest)

#Applying the Kuiper test

KuiperPGroupedDeg(sampletest, 72,1000)

kuiper.test(sampletest)

3. <u>Example for one of our simulations in R</u>

Script to simulate TB (Rao, HR, Gini) and Chi-squared test type 1 error of three sample sizes (10, 15 and 25)

#Loading libraries used in script

```
library(compiler)
library(circular)
library(NPCirc)
```

#setting options for script performance

```
options("scipen" = 10)
enableJIT(1)
```

#Set function for HR

```
HermansRasson2Tunc <- function(sample){
  n <- length(sample)
  total <- 0
  for (i in 1:n){
    for (j in 1:n){ total <- total + abs(abs(sample[i]-sample[j])-pi)-(pi/2)
    total <- total - (2.895*(abs(sin(sample[i]-sample[j]))-(2/pi)))}}
  T <- total/n
  return(T)}

HermansRasson2T <- cmpfun(HermansRasson2Tunc)
```

#Set function for Gini test

```
GiniTestValue <- function(sample){n <- length(sample)
f <- sort(sample)
fplus <- c(f[2:n],f[1])
D <- fplus - f
D[n] <- (2*pi) - f[n] + f[1]
nD <- n*D
Gn <- 0
for (i in 1:(n-1)){
  for (j in (i+1):n){Gn <- Gn + (0.5*abs(nD[i] - nD[j]))}}
Gn <- 2*Gn/(n*(n-1))
```

return(Gn)}

GiniTestValue <- cmpfun(GiniTestValue)HermansRasson2T <- cmpfun(HermansRasson2Tunc)

#Set function for Rao

```
RaoTestValue <- function(sample){n <- length(sample)
f <- sort(sample)
fplus <- c(f[2:n],f[1])
T <- fplus - f
T[n] <- (2*pi) - f[n] + f[1]
abs_diff <- abs(T-(2*pi/n))
U <- 0.5*sum(abs_diff)
return(U)}
```

#Set number of iterations, Sample size mean and kappa (or a sequence of any of that)

```
rans = 10000
nvseq = c(10,15,25)
l = length(nvseq)
mu=0
kappa=1000
```

#Prepare data frame for simulation results

```
Power.sample <- data.frame(matrix(nrow = l, ncol = 5))
names(Power.sample) <- c('RaoTB',"HRTB","GiniTB","RayleighTB","Chisquare")
#frequency table for Chi-squared
#make file for bins #expected prob
binsneeded <- rad(seq(0,350,10)+9.999); nullprob <- rep(1/36, 36)
```

# For-loop to run the tests on 10000 random distributions

```
for(e in 1:l){
  nv = nvseq[e]
  ###### set the distribution
  Distribution <- circular(sapply(1:rans, function(x) sample(rad(seq(0,360,10)),nv,replace = TRUE,prob = NULL)),units = 'radians')
```

```
### random error
errorsamp <- circular(sapply(1: rans, function(x)rvonmises(nv, mu, kappa)),units = 'radians') #calculating small Von mises error

######set random distributions with error added for Rao, HR and Gini
RandomSample <- circular(sapply(1:rans, function(x) sample(rad(seq(0,360,10)),nv,replace = TRUE,prob = NULL)),units = 'radians') # calculatating Distribution with m=36
RandomSample <- circular(RandomSample+errorsamp, units = 'radians') #adding error
RandomSample <- apply(RandomSample, 2, function(x) ifelse((x>(2*pi)),(x-(2*pi)),x)) #reducing modulo 2pi

###### apply the tests
##### adding error to Distribution
Distributionerror <-  circular(Distribution+errorsamp, units = 'radians')
Distributionerror <- apply(Distributionerror, 2, function(x) ifelse((x>(2*pi)),(x-(2*pi)),x)) #reducing modulo 2pi

RaoTest <- as.vector(apply(Distributionerror, 2, RaoTestValue)) # calculating Rao T
GiniTest <- as.vector(apply(Distributionerror, 2, GiniTestValue)) # calculating Gini
HRTest <- as.vector(apply(Distributionerror, 2, HermansRasson2T)) # calculating HRT
RhoTest <- as.vector(apply(Distributionerror, 2, rho.circular)) # calculating HRT

RandomU <-as.vector(apply(RandomSample,2, RaoTestValue)) #calcualting random Rao T
RaoSimP <- sapply(RaoTest, function(x) ((sum(1*(RandomU > x))/rans)))

RandomHR <-as.vector(apply(RandomSample,2, HermansRasson2T)) #calcualting random Rao T
HRT2p <- sapply(HRTest, function(x) ((sum(1*(RandomHR > x))/rans)))

RandomGini <-as.vector(apply(RandomSample,2, GiniTestValue)) #calcualting random Rao T
Ginip <- sapply(GiniTest, function(x) ((sum(1*(RandomGini > x))/rans)))
```

```
  RandomRho     <-as.vector(apply(RandomSample,2,    rho.circular))    #calcualting
random Rho
  RayP <- sapply(RhoTest, function(x) ((sum(1*(RandomRho > x))/rans)))

  #####Calculate p-value and store in Power.sample file
  Power.sample$RaoTB[e]<- sum(1*(RaoSimP < 0.05))/rans
  Power.sample$HRTB[e]<- sum(1*(HRT2p < 0.05))/rans
  Power.sample$GiniTB[e]<- sum(1*(Ginip < 0.05))/rans
  Power.sample$RayleighTB[e]<- sum(1*(RayP< 0.05))/rans

  ######Chi-squared
  ######bin the data
  Distributionbinned1    <-    apply(Distribution,2,function(x)    as.vector(summary(cut(x,
breaks = binsneeded, right = F, include.lowest = T))))
  Distributionbinned <- array(as.matrix(unlist(Distributionbinned1)), dim=c(36, rans))

  ###### apply the test
  chisquarelist<- apply(Distributionbinned,2,function(x) chisq.test(x,p=nullprob))
  ChisquareP <- sapply(c(1:rans),function(x) chisquarelist[[x]][["p.value"]])

  #####Calculate p-value and store in Power.sample file
  Power.sample$Chisquare[e]<- sum(1*(ChisquareP < 0.05))/rans
} #e

Type_1_error <- data.frame(nvseq,Power.sample )
```
